# Auditory feedback modulates development of kitten vocalizations

**DOI:** 10.1007/s00441-014-2059-6

**Published:** 2014-12-19

**Authors:** Peter Hubka, Wiebke Konerding, Andrej Kral

**Affiliations:** 1 Institute of AudioNeuroTechnology and Department of Experimental Otology, ENT Clinics, Cluster of Excellence ‘Hearing4all’, Hannover Medical School, Feodor-Lynen-Str. 35, 30175 Hannover, Germany; 2 School of Behavioral and Brain Sciences, The University of Texas at Dallas, Richardson, TX USA

**Keywords:** Deafness, Congenitally deaf white cat, Auditory, Deprivation, Hearing loss

## Abstract

Effects of hearing loss on vocal behavior are species-specific. To study the impact of auditory feedback on feline vocal behavior, vocalizations of normal-hearing, hearing-impaired (white) and congenitally deaf (white) cats were analyzed at around weaning age. Eleven animals were placed in a soundproof booth for 30 min at different ages, from the first to the beginning of the fourth postnatal month, every 2 weeks of life. In total, 13,874 vocalizations were analyzed using an automated procedure. Firstly, vocalizations were detected and segmented, with voiced and unvoiced vocalizations being differentiated. The voiced isolation calls (‘meow’) were further analyzed. These vocalizations showed developmental changes affecting several parameters in hearing controls, whereas the developmental sequence was delayed in congenitally deaf cats. In hearing-impaired and deaf animals, we observed differences both in vocal behavior (loudness and duration) and in the calls’ acoustic structure (fundamental frequency and higher harmonics). The fundamental frequency decreased with age in all groups, most likely due to maturation of the vocal apparatus. In deaf cats, however, other aspects of the acoustic structure of the vocalizations did not fully mature. The harmonic ratio (i.e., frequency of first harmonic divided by fundamental frequency) was higher and more variable in deaf cats than in the other study groups. Auditory feedback thus affects the acoustic structure of vocalizations and their ontogenetic development. The study suggests that both the vocal apparatus and its neuronal motor control are subject to maturational processes, whereas the latter is additionally dependent on auditory feedback in cats.

## Introduction

Congenital deafness affects the development of the auditory system (Kral and Sharma 2012; Kral 2013) and impacts a number of non-auditory functions, including reading, fine motor coordination, attention, working memory, executive function, sequence learning and others (Myklebust 1960; Horn et al. 2006; Dye et al. 2009; Conway et al. 2009; 2011; Kral and O'Donoghue 2010; Kronenberger et al. 2013). Disturbed auditory feedback influences vocalization behavior, variability and maintenance of the vocal structure in species demonstrating vocal learning (Marler and Waser 1977; Leonardo and Konishi 1999; Woolley and Rubel 1997; Nagel et al. 2011; Rajan and Doupe 2013). However, vocal non-learners as a rule do not show a significant influence of the hearing status on vocalizations (Hammerschmidt et al. 2012; Mahrt et al. 2013).

To determine how congenital deafness affects vocal control in the cat, the present study investigates vocalization behavior and the acoustic structure of vocalizations in cats with mild and profound hearing loss. Cats use a set of vocalizations that are produced in distinct behavioral contexts (Moelk 1944; Brown et al. 1978; Nicastro and Owren 2003). Feline vocalizations are often considered automatic behavioral programs that are elicited in brainstem nuclei (Holstege 1989; van der Horst and Holstege 1996). These nuclei are under the influence of periaqueductal gray, reticular formation and amygdala, septum, basal ganglia and hypothalamus (Altafullah et al. 1988), whereas only the lower levels (periaqueductal gray and below) appear necessary for spontaneous vocalizations (Skultety 1958; Jürgens and Pratt 1979; Ploog 1981; Zhang et al. 1992; Ackermann et al. 2014; cf. Arriaga and Jarvis 2013). Animal vocalizations are phonetic precursors of language; however, they differ from language in many respects (Arbib 2005), particularly regarding the absence of the symbolic nature of communication (Hauser et al. 2002). Nonetheless, primate vocalizations appear to be at least partially under the influence of frontal cortical areas (Roy et al. 2011; Hage and Nieder 2013). Recently, direct sparse connections between the motor cortex and the brainstem nuclei responsible for vocalizations were demonstrated in mice, a species considered a vocal non-learner (Arriaga et al. 2012). Furthermore, some effects of hearing loss on vocal behavior have been demonstrated in the same species (Arriaga et al. 2012). This supports a more gradual transition in vocal behavior between vocal learners and non-learners, as expressed in the continuum hypothesis (Arriaga and Jarvis 2013; Petkov and Jarvis 2012).

Due to their important social role, vocalizations represent a cardinal stimulus for the brain. Neurons in many auditory structures of animals respond strongly to the specific time and frequency structure of vocalizations (Gehr et al. 2000; Gourévitch and Eggermont 2007; Carrasco and Lomber 2011; for primates, see Wang and Kadia 2001; Eliades and Wang 2008; Romanski and Averbeck 2009; Romanski 2012). Modification of the vocal apparatus and thus of acoustic properties of vocalizations, affects the developmental responsiveness of cortical neurons (Cheung et al. 2005). This demonstrates that the auditory cortex also adapts to the individual’s own vocal production.

Vocalizations themselves also undergo substantial developmental changes during the postnatal period. These developmental changes are determined by peripheral factors (including anatomical development of the vocal cord and vocal tract; Sato and Hirano 1997; Sato et al. 2001; Ward et al. 2002) and central factors (maturation of the central nervous system). Whereas the peripheral factors related to anatomical changes are independent of the subject’s hearing, the central neuronal factors may be influenced by auditory feedback. The role of this influence during postnatal development is the focus of the present study.

White blue-eyed cats are known to have a higher incidence of deafness than other cats (Bosher and Hallpike 1965; Mair and Elverland 1977; Heid et al. 1998; Ryugo et al. 1998). The deaf animals, selected from the colony by a hearing-screening procedure (Heid et al. 1998), have no hearing experience due to an inherited degeneration of the organ of Corti before the onset of hearing (Mair and Elverland 1977; Heid et al. 1998). They are thus congenitally deaf. Other animals from the same colony may have their hearing impaired to different degrees (Heid et al. 1998; Geigy et al. 2007). The cortical developmental sequence of congenitally deaf cats (CDCs), compared to normal-hearing cats, revealed developmental delays and alterations (Kral et al. 2005; Kral and Sharma 2012). When the CDCs received cochlear implants early in life and were stimulated electrically over 2–5 months, many of the deficits were compensated for, with feature sensitivity in the auditory cortex improved (Kral et al. 2006, 2013b). There are sensitive periods for such maturational effects of cochlear implants (Kral et al. 2006, 2013a, b; Kral and Sharma 2012). Consequently, the effects of hearing experience in cats provide an explanation of neuronal mechanisms of adaptation to cochlear implants in prelingually deaf children (Kral and Sharma 2012).

Previous studies on effects of hearing loss on vocalizations in cats provide an equivocal picture: in some investigations, only limited effects of hearing loss on vocalizations have been observed (see e.g. Talmage-Riggs et al. 1972), whereas other authors have reported differences in some of the parameters of the vocalizations (Romand and Ehret 1984; Shipley et al. 1988). Very substantial cortical development is observed in the first 1.5–3.0 months after birth in cats (Eggermont 1996; Kral et al. 2005). The functional role of feline vocalizations potentially changes during this time (Brown et al. 1978; Ehret 1980; Turner and Bateson 2000). Therefore, this developmental period appears of cardinal importance for investigation of the role of central neuronal maturation in vocalizations.

The present study statistically compares vocalizations of normal-hearing, hearing-impaired and deaf kittens during the first 3 months of life. The investigated call type (a voiced vocalization denoted the ‘isolation call’) shares some common features with vowels of human language and has been suggested to be similar in structure and function to the cry human infants generate under social isolation (Newman 1985, 2007). The study shows that, despite the general presence of isolation calls in all congenitally deaf cats, vocal behavior (as measured by loudness and duration of vocalization) is not the only difference between deaf and hearing animals: the details of the acoustic structure of the isolation call and their variability within a recording session, were also affected by deafness. Finally, complete deafness had a more pronounced effect on vocalizations than did partial hearing impairment.

## Materials and methods

In the present behavioral experiments, four normal-hearing mongrel cats, four hearing-impaired white cats and three congenitally deaf cats (CDCs) were used. All hearing-impaired and deaf animals were drawn from a colony of white cats. Hearing status was assessed during a screening procedure at the age of 4 weeks postnatally (p.n.; Heid et al. 1998). This objective assessment of hearing is described in detail elsewhere (Heid et al. 1998; Tillein et al. 2012); it was performed in sedated animals using recordings of auditory-evoked brainstem responses with condensation clicks (50 μs duration). Normal-hearing animals had the lowest hearing sensitivity (≤40 dB SPL) and deaf animals showed no responses up to 110 dB SPL in both ears. Animals from the white cat colony with mild hearing loss (hearing loss <40 dB) were used for further comparisons (see “Results”).

Vocalizations were triggered by isolating the animal from its mother and siblings and placing it in a soundproof booth (double-wall anechoic chamber; Industrial Acoustics, Germany). To prevent exploration, the available space for the animal was further limited by an acoustically transparent cage (dimensions 45 × 30 × 30 cm). The calibrated microphone (Bruel & Kjaer 4165 condenser ½” microphone with a Bruel & Kjaer 2209 amplifier) was positioned in front of the cage at a distance of 50 cm. The situation reliably induced spontaneous vocalizations that were recorded continuously for 30 min, sampled at 44.1 kHz and stored on a computer. The data were analyzed offline using custom-tailored software programmed in MATLAB (©Mathworks) (Kraschon et al. 2007).

Vocalizations were recorded between days 30 and 120 after birth (p.n.), with a total of 13–15 recording sessions in each animal group. The data were subsequently pooled into four age categories: 1 month (days 30–35 p.n.), 1.5 months (days 36–42 p.n.), 2 months (days 44–58 p.n.) and 3 months (days 64–103 p.n.). This corresponds to the developmental timeline of the auditory brainstem (Tillein et al. 2012) and covers the developmental stage with the most pronounced difference in brain development between deaf and hearing cats (Ehret 1980; Kral et al. 2005).

### Signal analysis

The recorded data were subsequently analyzed by an objective procedure programmed in MATLAB (Kraschon et al. 2007). Firstly, the signals were high-pass filtered to remove low-frequency components not contained in cat isolation calls (Butterworth filter, 10th order, 500 Hz high-pass). Afterwards, a procedure for detecting sounds exceeding the background noise level was used as follows: a sliding window of 90 ms duration was moved over the entire 30-min signal and, within this window, the root-mean-square of the signal was computed. Of the minimum value obtained within a given session, 130 % was used as a threshold value to detect vocalizations in this session. The procedure was manually tested for robustness.

To track qualitative developmental changes in vocalizations, the spectrogram representations of individual vocalizations were averaged, resulting in an average spectrogram per session in each cat. For this purpose, spectrograms were first computed in MATLAB using short-time Fourier transform (Hamming window, FFT length of 1024, overlap of 75 %). Individual spectrograms were then aligned to the onset of vocalization and normalized to the maximum amplitude within the individual spectrogram. The normalized aligned spectrograms were averaged. Onset alignment enabled us to track the changing frequency components from onset of vocalization onwards. Overall representation of frequency content of vocalization (power spectra) was additionally evaluated using the Welch method (FFT length of 1024). The power spectra, too, were first normalized to the maximum power and subsequently averaged. To assure that the onset part of the vocalization was well represented in the average spectrogram, vocalizations that contained multiple unvoiced components (unvoiced or combined vocalizations) were excluded from the construction of average spectrograms, as were vocalizations shorter than 100 ms or longer than 1,500 ms.

For further quantitative analysis, 10 ms non-overlapping frames were used to differentiate voiced and voiceless segments (cf. Markel 1972). Voicing was detected using autocorrelation on the detected segments. If the ratio of the maximum autocorrelation coefficient in the delay range exceeded 0 ms and its value at 0 ms delay exceeded a threshold of 0.6, the segment was considered voiced. For classification of the entire call, algorithmic corrections based on previous segments were applied using the simple inverse filter tracking method (Markel 1972).

The fundamental frequency (F0) of voiced vocalizations was determined from the autocorrelation function; to avoid confusion with first harmonics, the robustness of the results was increased by limiting the frequency range to around ±30 % of the expected maximum frequency of F0 (Manfredi et al. 2000; Manfredi 2006). The outcome of the procedure was additionally manually controlled to avoid ‘contamination’ by imprecise identification of the fundamental frequency. For these (and the following) purposes, a time-frequency analysis was performed using fast Fourier analysis with a 10-ms sliding window and 50 % overlap and the results were plotted as normalized power density. For visual control, 5-ms windows with 97 % overlap were also used. The time-frequency representation was smoothed using a 5-point median filter that did not change the position of the energy maxima in this representation. F0 was characterized by its mean, maximum and standard deviation (a measure of its variability). The time of maximum F0 was additionally determined for each call and processed the same way. The F1/F0 harmonic ratio was also assessed, defined by its mean, standard deviation, maximum and the time of its maximal value (which were additionally determined).

The data’s normality was tested using the Stephens–Pearson test (α = 10 %) and the data were compared using *F* tests (for variance equality) and *t* tests (two-tailed uncorrected). The significance level was 5 % in all cases.

## Results

The present data were obtained from three groups of animals: hearing controls from a colony of normal-hearing cats, animals from the white-cat colony with well-preserved hearing (hearing-impaired animals) and congenitally deaf cats from the white-cat colony (congenitally deaf cats, CDCs). The hearing screening (Heid et al. 1998; Tillein et al. 2012), performed at 1 month after birth, revealed that the normal-hearing kittens had a mean ABR threshold (mean for both ears and all animals) of 31 ± 2.2 dB SPL, whereas the hearing-impaired animals had a mean threshold of 43.3 ± 4.9 dB SPL. In the deaf cats, we could not detect any responses up to 110 dB SPL. Under the clinical classification (Kral and O'Donoghue 2010), therefore, the hearing-impaired animals would be identified as having mild hearing loss and the CDCs as being profoundly deaf. For the present results, it is important that the impaired group had only mildly elevated thresholds compared with hearing controls.

In total, we recorded 13,864 vocalizations (hearing: 4,757; hearing impaired: 3,718; deaf: 5,389). For the vocalization identification and segmentation procedure, a subset of 3,508 vocalizations (obtained from all animal groups) was manually post-processed and compared with the results of the automatic segmentation procedure. The mean error rate per processed recording was 2.73 %, with the maximum value observed at 5.8 %. As a rule, more vocalizations were identified by the automatic procedure than manually, the reason being inappropriate ‘splitting’ of some vocalizations into two calls during automatic segmentation. Because of these low error rates, we used the automatic procedure for further processing.

**Fig. 1:**
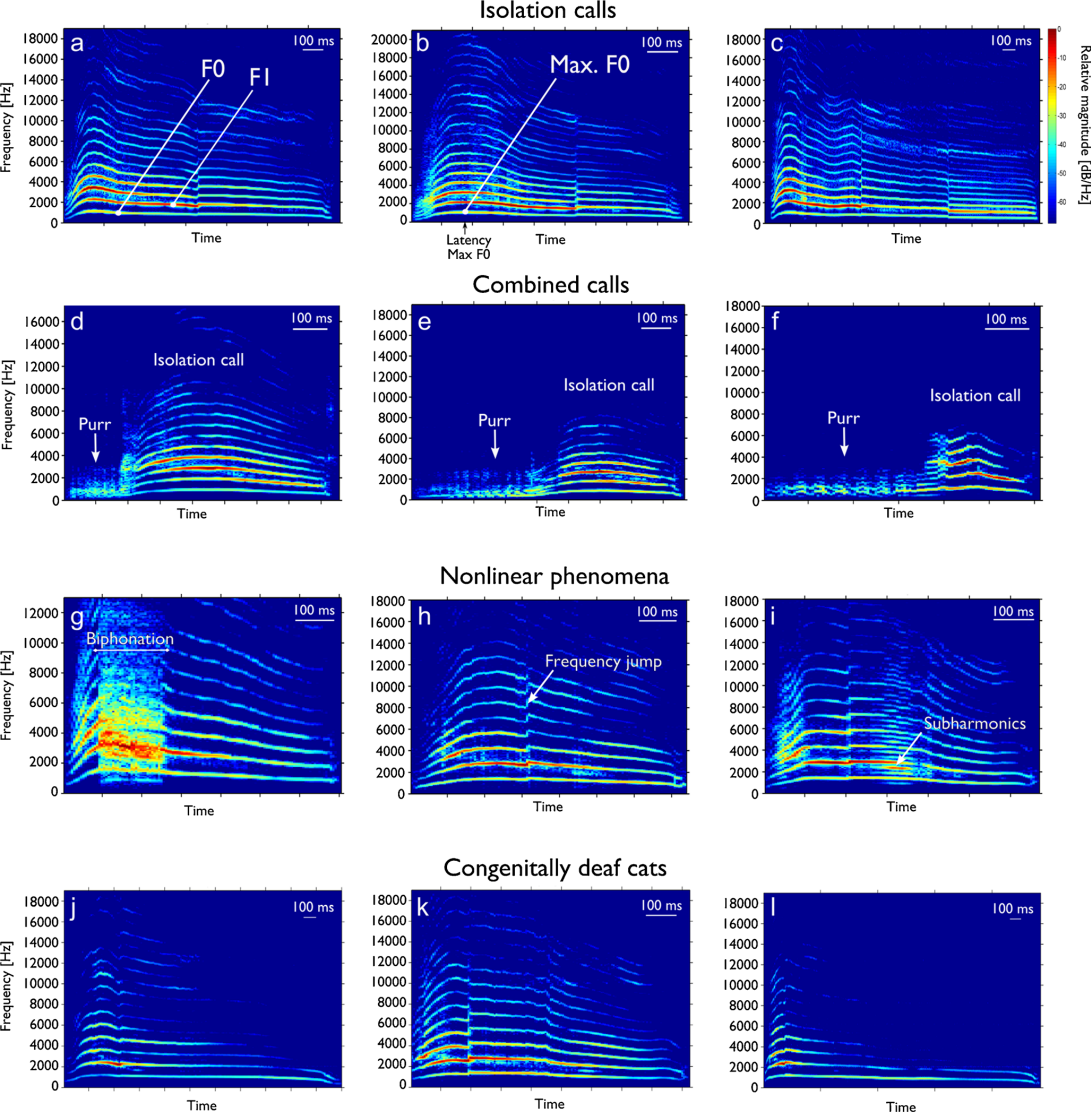
Example spectrograms of isolation and combined calls. **a**–**c** Isolation calls are characterized by a harmonic structure. **b** The louder calls cover the entire frequency range accessible for analysis. **c** The isolation calls can vary in duration and in total may reach 2 s. Quantification of isolation calls was performed for F0, F1, their ratio and the maximum F0. **d**–**f** Combined calls were excluded from analysis; usually they started as an unvoiced call and, after a certain (varying) time, changed into a voiced call. **g**–**i**: Non-linear phenomena were often found in the calls. **g** A biphonation is characterized by a temporal breakdown of the harmonic structure of the call. **h** Frequency jumps were frequent in isolation calls (see also (**a**–**c**)). **i** Subharmonics are characterized by occurrence of additional components in the harmonic structure. **j**–**l** Examples of vocalizations from a congenitally deaf cat

Vocalizations were first automatically classified as voiced and unvoiced, as described above. In all animal groups, the vast majority of the vocalizations were voiced, whereas hearing-impaired animals showed significantly fewer voiced vocalizations than did the other two groups of animals (hearing: 89 ± 6 %; deaf: 86.6 ± 9.8 %; impaired 69.7 ± 24.5 %; two-tailed *t* test, hearing vs. deaf: *p* = 0.442; hearing vs. impaired: *p* = 0.0096; impaired vs. deaf: *p* = 0.0025). This relation was similar across all age groups. The voiced vocalizations were identified as isolation calls based on the overall acoustic structure (Fig. 1a). The lowest frequency of the spectrogram, called the fundamental frequency (F0), was near 1 kHz. The spectrogram was further characterized by harmonically related frequency components, whereas for quantification purposes the first harmonic frequency was used, here denoted F1 (Fig. 1a). The maximum energy was observed either at the first or the second harmonic-frequency component. The isolation calls showed high intra-individual variability (Fig. 1a–i). The variability of the calls was expressed in several features including call durations – see Fig. 1a-c – and differently steep frequency increases at the onset (and thus the time at which the maximum F0 was reached). The frequency range of isolation calls, if of sufficient loudness, covered the whole frequency range available for analysis (500–22,000 Hz for f_s_ = 44.1 kHz) in all experimental groups (Fig. 1b). The isolation calls were characterized as having the greatest energy at F1 or F2 and call durations of a few hundred ms up to ∼2 s (Fig. 1).

Unvoiced vocalizations (excluded from further analysis) were mainly purr-like vocalizations and combined calls, starting as an unvoiced call and continuing as an isolation call (Fig. 1d–f). The duration of the individual components differed in individual behavior with regard to combined calls. Some isolation calls contained biphonations, frequency jumps or subharmonics (Fig. 1g–i) which, to the listener, sometimes sounded as if they were conveying extra emotional emphasis (Nicastro and Owren 2003). These could appear at different latencies within the call (Fig. 1g–i).

Congenitally deaf cats also produced isolation calls (Fig. 1j–l). Manual inspection of the vocalizations of CDCs also revealed high variability in individual calling behavior. All typical acoustic signs of isolation calls were observed in CDCs. This indicates the overall stability of the vocalizations and their general presence even in total absence of hearing. However, experienced animal caretakers reported that deaf cats had calls that sounded sharper and slightly different from hearing controls.

**Fig. 2:**
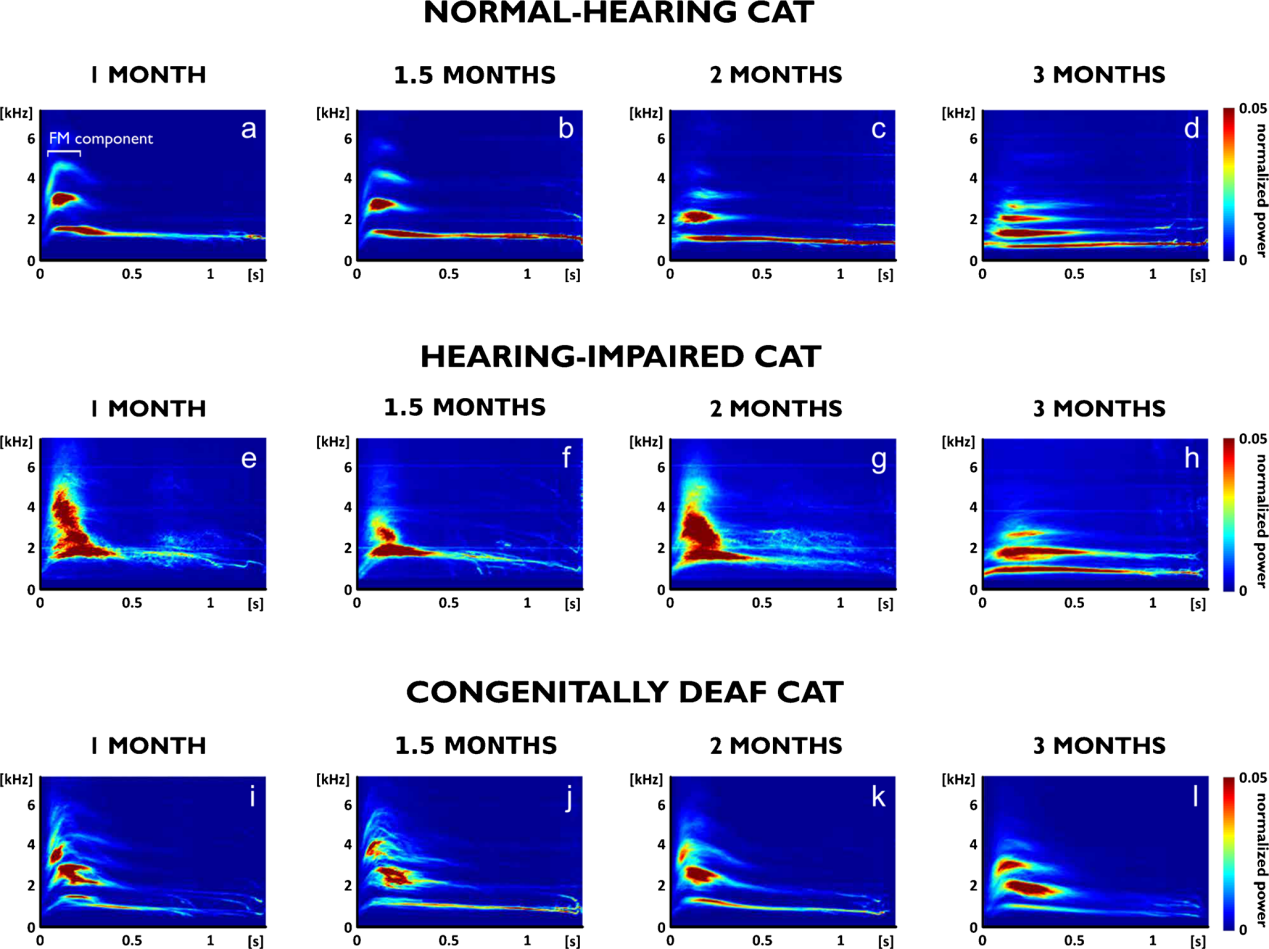
Development of mean spectrogram representations in the three experimental groups, obtained as an average of all voiced vocalizations at the given age in a representative animal. **a**–**d** In normal-hearing cats, the mean spectrograms reveal the harmonic structure of the call and a developmental change in the onset upward/downward FM sweep that flattens with increasing age (**c**, **d**). Furthermore, the dominant frequencies decrease with age. **e**–**h** In the hearing-impaired group, the mean spectrograms show a smeared higher harmonic structure (compare **e**–**g** to **a**–**c**), indicating greater variability in this spectral range. However, at 3 months (**h**) a mature pattern of vocalization was also observable in this group. **i**-**j**: In congenitally deaf cats, the dominant frequencies decrease with age; however, the FM sweep at onset of vocalization is present also at 3 months (**l**), indicating a decelerated or arrested developmental sequence in this respect.

The spectrograms of the vocalizations of each animal were amplitude-normalized to maximum, aligned to the onset of vocalization and averaged for each age group (Fig. 2). These average spectrograms revealed a developmental sequence in the spectral structure of hearing animals: at 1 month p.n., the onsets of the vocalizations contained an upward/downward FM component (Fig. 2a). This characterized a juvenile acoustic structure of the kitten isolation call (see Brown et al. 1978). In hearing controls, the F0 decreased with increasing age and the initial FM component was substantially reduced, so that at 2 months p.n. the juvenile characteristics were no longer apparent (Fig. 2c, d). Hearing-impaired animals, on the other hand, had a less stable frequency structure, resulting in smeared higher harmonics in the average spectrogram (Fig. 2e). At the age of 3 months, however, the vocalizations were well comparable with hearing controls of the same age (Fig. 2h). This was not the case with the congenitally deaf cats: despite decreasing F0 with increasing age, the deaf cats still had discernible onset FM components even in the oldest age group (Fig. 2l). This demonstrates delayed or absent development of the acoustic structure of vocalizations, with a persistent juvenile character of the isolation calls at 3 months in CDCs.

**Fig. 3:**
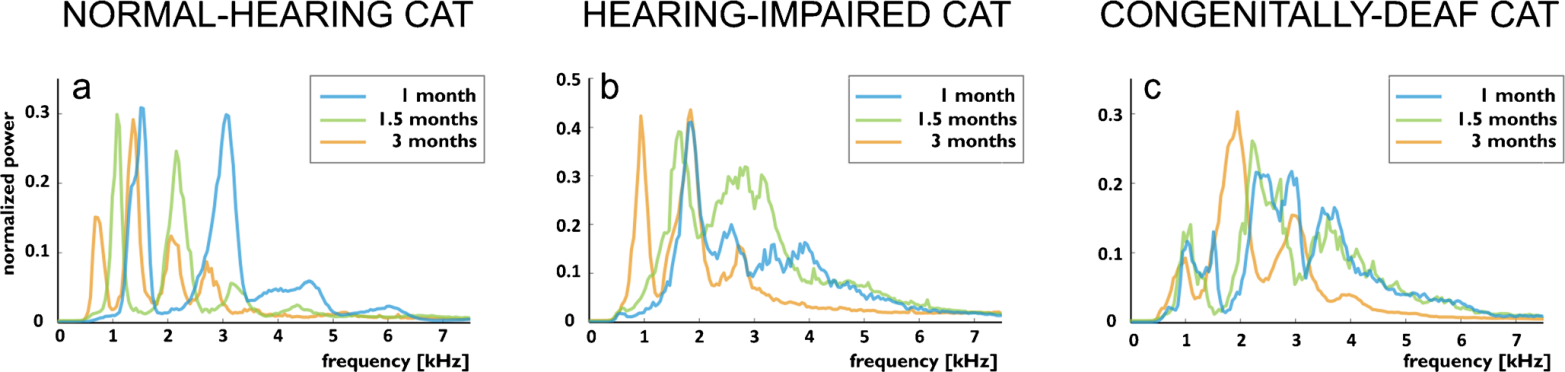
Power spectra of the voiced vocalizations, shown as mean values for all vocalizations in a representative single animal during development. To improve the discernibility of the spectra, the spectrum at the age of 2 months is not shown. **a** In normal-hearing cats, a decrease in dominant frequencies with age is observable. Distinct and sharp peaks in the mean spectrum show up. The variability of dominant frequencies within each age group is consequently small. **b** In hearing-impaired animals, a decrease in frequencies is also observable; however, the peaks are broader. **c** The congenitally deaf cats exhibited the broadest peaks, demonstrating a large variability of calls

Furthermore, in the assessment of the vocalizations’ harmonic structure, the mean power spectra of the entire call (Fig. 3) confirmed that—corresponding to decreasing F0—the higher harmonics decreased with increasing age in all three groups of animals. In comparison with normal-hearing animals, the deaf cats showed a wider power distribution around the spectral peaks, indicating higher variability in the spectral structure of the calls.

To statistically confirm the results of Figs. 2 and 3, quantitative processing of each individual vocalization from all animals was automatically performed and the data were statistically compared for different acoustic features of the isolation call.

## Vocal behavior

**Fig. 4:**
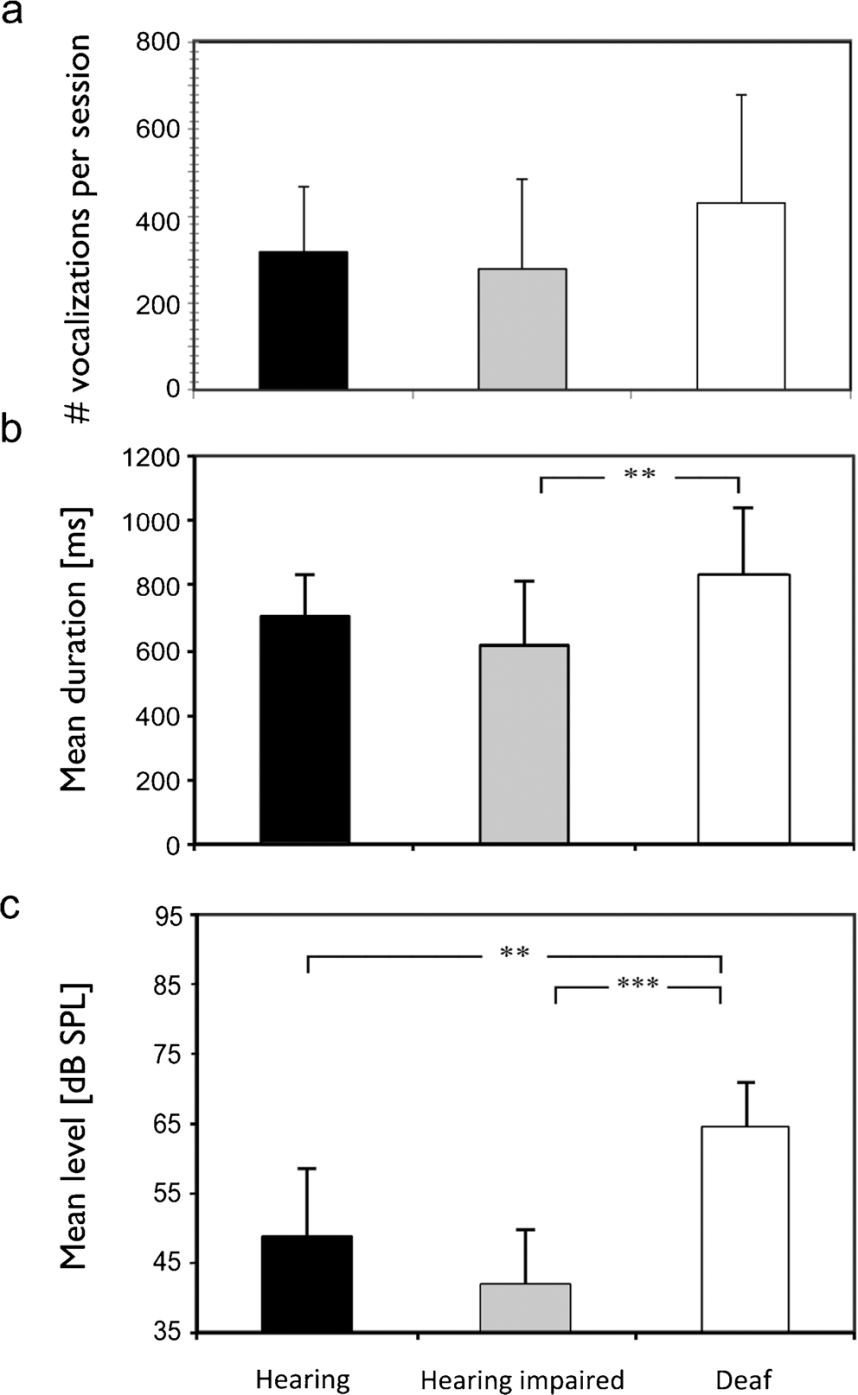
Comparison of basic characteristics of vocalizations. **a** Number of vocalizations per session with no significant difference between hearing, hearing-impaired white cats and congenitally deaf (white) cats. **b** Mean vocalization duration showed significant effects only between hearing-impaired and deaf cats. **c** Mean level of vocalizations was significantly higher in deaf cats than in all other animals. Two-tailed *t* test. **∼ *p* < 0.01; ***∼ *p* < 0.001

First, the general properties of the vocalizations were analyzed groupwise for all ages pooled, with means calculated per session; these were then pooled irrespective of age, resulting in a grand mean. The animals vocalized quite often in the soundproof booth: they generated 200–600 vocalizations per session (Fig. 4a). This high frequency of vocalization was attributable to the unfamiliar environment, the social separation and the limited space in the cage. There was no difference in the rate of vocalizations between the animal groups (hearing vs. impaired: *p* = 0.557; hearing vs. deaf: *p* = 0.172; impaired vs. deaf: *p* = 0.09). This was still evident when comparisons were performed within one age group (all *p* > 0.05). However, the deaf animals tended to exhibit the longest vocalizations (Fig. 4b; hearing vs. impaired: *p* = 0.165; hearing vs. deaf: *p* = 0.063; impaired vs. deaf: *p* = 0.0094).

The sound-pressure level (SPL) of the vocalizations was additionally analyzed; however, these results have to be considered with caution, as the absolute SPL picked up by the microphone critically depends on the relative position of the animal and the microphone. As the animals were unrestrained within the cage, this position was variable. Call loudness was significantly higher in deaf cats (Fig. 4c). The vocalizations were ∼10 dB louder than in hearing and hearing-impaired animals (hearing vs. impaired: *p* = 0.184; hearing vs. deaf: *p* = 0.0011; impaired vs. deaf: *p* = 0.0002). This is interesting since, combined with the tendency for longer duration in deaf cats, the effects on the overall energy of the calls are even greater.

**Fig. 5:**
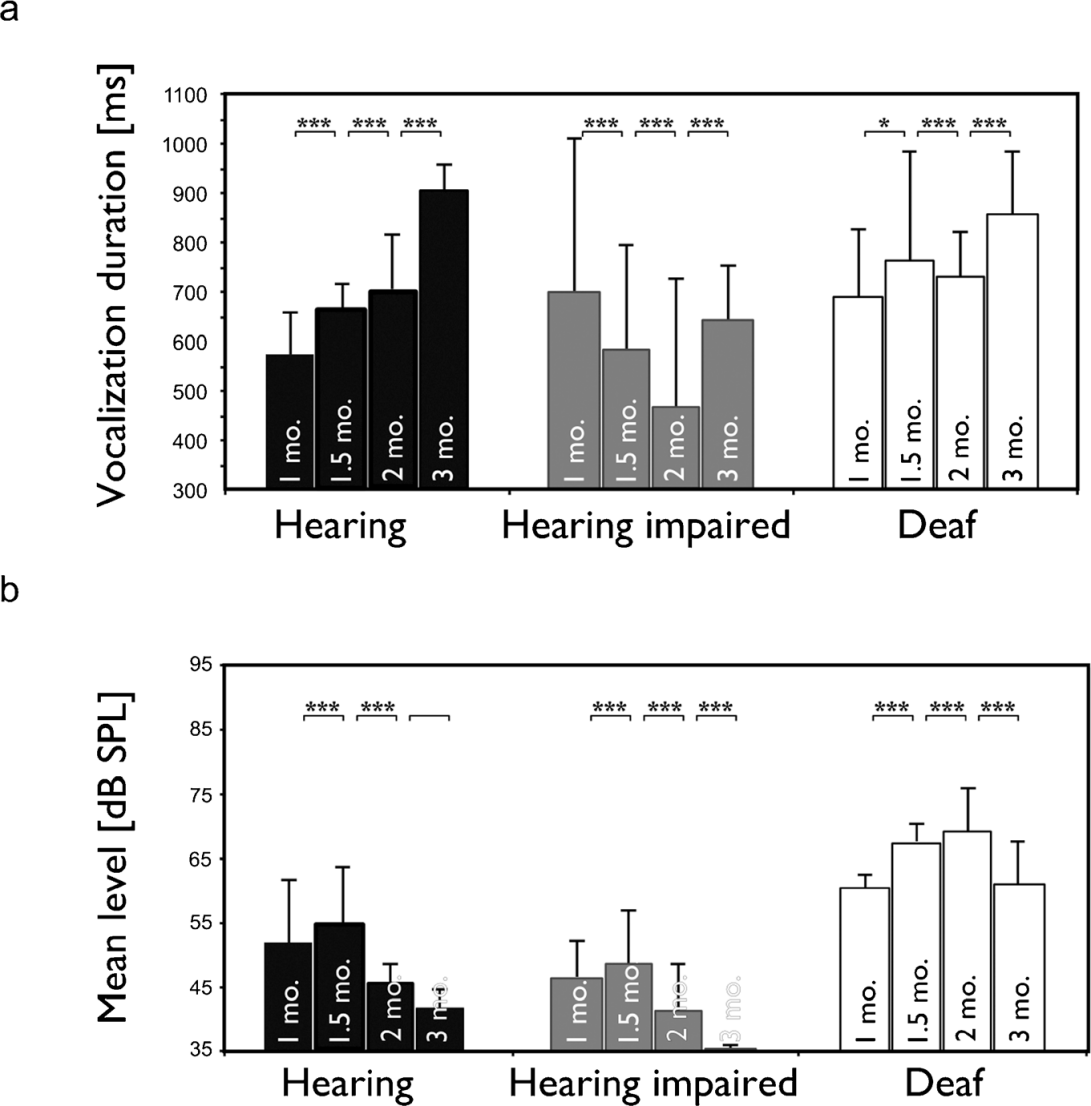
Developmental changes in vocalization characteristics show an increase in vocalization duration with age in hearing controls (**a**) but no systematic change in hearing- impaired and deaf cats. The mean level decreased systematically from 1.5 months onwards in normal-hearing and hearing-impaired animals but deaf cats demonstrated a different developmental pattern (**b**). Two-tailed *t* test. **∼ *p* < 0.01; ***∼ *p* < 0.001

Next, the developmental pattern of the call properties was analyzed. This involved generating age-related statistics within groups. The number of vocalizations did not differ between the three investigated groups at any age (data not shown; *p* > 0.05). However, the developmental pattern of vocalization duration differed between the groups. Hearing animals showed increasing call duration with increasing age (Fig. 5a). None of the other groups exhibited such a pattern; rather, they showed nonsystematic variation in duration with age. This indicates that hearing loss has affected the developmental sequence of vocalization behavior. Interestingly, hearing kittens exhibited shorter vocalizations than deaf cats at 1 and 1.5 months (*p* < 0.001). The difference, however, was not significant at 2 months (*p* = 0.103) and reversed at 3 months, when hearing cats had longer vocalizations than deaf cats (*p* < 0.001).

The developmental sequence also differed with regard to the loudness of vocalizations. In all age groups, the loudest vocalizations were found in deaf animals (*p* < 0.001) and the faintest in hearing-impaired animals (*p* < 0.001), whereas those of both the hearing and hearing-impaired animals first increased and then decreased with increasing age (Fig. 5b). CDCs did not display a systematic change with age (Fig. 5b), indicating arrested or delayed development.

## Vocalization structure

**Fig. 6:**
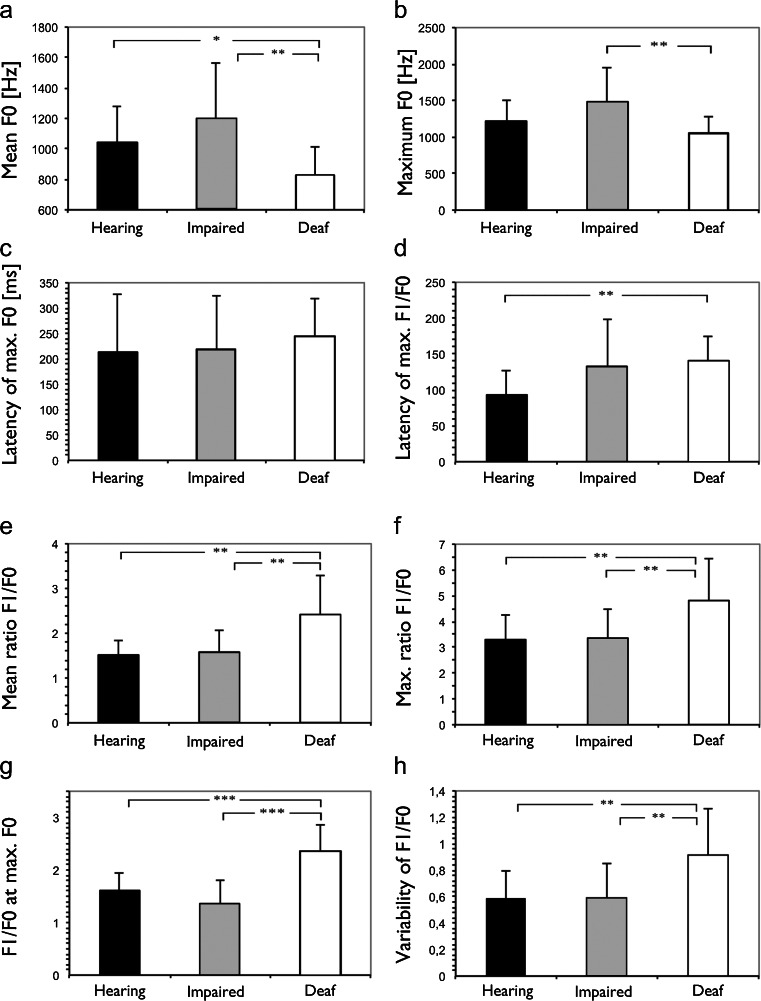
Analysis of the structure of individual vocalizations. **a** Fundamental frequency was lower in deaf cats. **b** The maximum fundamental frequency differed only between hearing-impaired and deaf cats. **c** The latency from vocalization onset when the maximum F0 is reached was higher in deaf cats. **d** The latency from onset of vocalization when the maximum harmonic ratio was reached was longer in deaf cats. **e** Harmonic ratio was highest in deaf cats and hearing-impaired and normal-hearing cats did not differ with regard to this measure. Furthermore, the maximum harmonic ratio (**f**) and the harmonic ratio at the maximum F0 (**g**) was highest in deaf cats. Finally, the harmonic ratio (quantified as standard deviation) was more variable in deaf cats (**h**). Two-tailed *t* test. *∼ *p* < 0.05; **∼ *p* < 0.01; ***∼ *p* < 0.001

To analyze whether also the acoustic structure was changed by absence of hearing, several parameters were quantified in the isolation calls, starting with mean F0, maximum F0 and latency of maximum of F0. Firstly, the grand means from all sessions were compared once again. Mean F0 was significantly smaller in deaf cats than in the other groups (Fig. 6a), whereas the difference in maximum F0 was less well expressed (Fig. 6b). The time (i.e., latency) of the maximum value of F0 did not differ between the animal groups (Fig. 6c). Since developmental changes in F0 may be a simple reflection of the anatomical situation in the vocalization apparatus (i.e., growth during development), they are mainly determined by maturation of peripheral factors. For this reason, the harmonic ratio, i.e., F1/F0, was additionally calculated and analyzed. This should be less affected by the growth of the vocalization apparatus. Congenitally deaf cats had a larger latency of maximal harmonic ratio (Fig. 6d). Furthermore, both the mean and the maximum F1/F0 harmonic ratio of the hearing-impaired animals was not significantly different from hearing controls but the deaf cats had a significantly higher harmonic ratio (Fig. 6e, f). The harmonic ratio at the time of maximum F0 was also larger in deaf cats (Fig. 6g). Finally, we noted a significant increase in the variability of vocalizations in deaf cats, reflected in the greater variability of the harmonic ratio (Fig. 6h). Altogether, in terms of harmonic ratio, the deaf cats significantly differed from the other two groups, whereas hearing-impaired animals were similar to hearing controls.

**Fig. 7:**
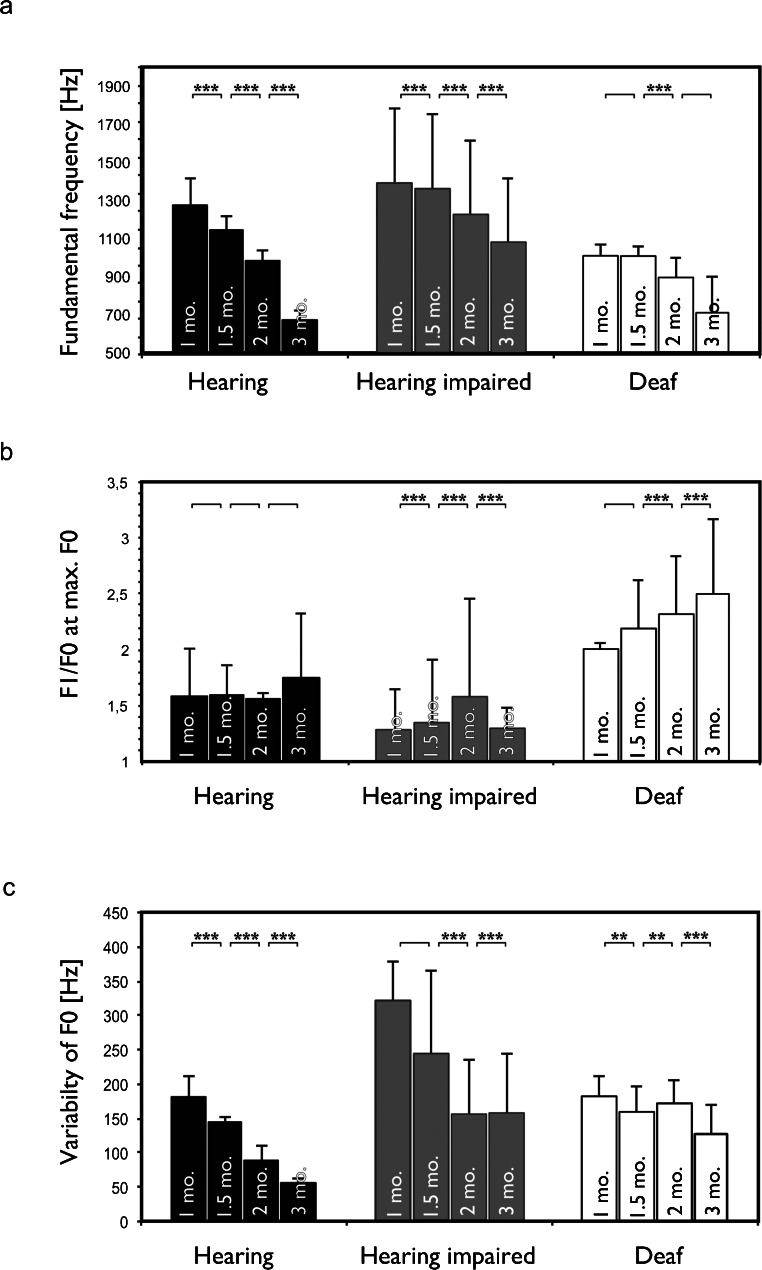
**a** Developmental changes in fundamental frequency exhibit a consistent decrease with age in all three groups of animals. This is likely related to changes in size of the vocal apparatus. **b** Harmonic ratio did not demonstrate any changes with age in hearing controls but a consistent increase with age in deaf animals. **c** Consistent with a decreasing fundamental frequency, the variability of F0 showed a decrease with age in hearing controls but the changes were less pronounced in deaf cats. Two-tailed *t* test. **∼ *p* < 0.01; ***∼ *p* < 0.001

Developmental data were analyzed next and are presented for those parameters where a systematic change with age could be observed in at least one group of animals. With increasing age, F0 decreased significantly in hearing and hearing-impaired cats (Fig. 7a). This change was less pronounced in deaf cats. As expected, the F1/F0 harmonic ratio was not systematically affected by age in hearing controls and hearing-impaired animals (Fig. 7b). In the group of deaf cats, however, the harmonic ratio increased with increasing age (Fig. 7b). This demonstrates the consistently abnormal development of isolation calls in deaf animals.

Finally, the variability of F0, measured by its standard deviation, decreased with increasing age in hearing controls, whereas the developmental sequence was not obvious in the hearing-impaired and the deaf cats (Fig. 7c). This finding suggests that auditory feedback has a role in stabilizing the acoustic structure of the call.

## Discussion

Using a large dataset, the present study demonstrates that hearing affects the development and the properties of isolation calls in the cat. For the first time in the cat, an analysis of this kind was performed using an automated procedure custom-designed for this task. Furthermore, this is the first time a study was conducted on the effects of both mild and profound hearing loss on vocalizations, allowing comparison between the effects of normal, altered and absent auditory feedback.

In deaf cats, the development of the calls’ acoustic structure was delayed or incomplete. Several aspects of the development of this acoustic structure in hearing-impaired animals were similar to that in hearing controls. Nevertheless, they also exhibited a less stable (i.e., more variable) spectral structure than in hearing controls. This indicates a graded effect of mild to profound hearing loss based on the degree of auditory feedback.

Consequently cat vocalizations are not solely produced by automated neuronal programs but partially depend on feedback and show developmental plasticity. A prominent finding was the greater variability of the spectral properties of vocalizations in deaf cats. Therefore, auditory feedback is used to control and stabilize the vocalizations during development in hearing cats.

### Methodological discussion

Previous studies on feline vocalizations documented about 10 different vocalizations generated in different behavioral contexts (Moelk 1944; Brown et al. 1978; Farley et al. 1992). The present study focused on isolation calls (Fig. 1a–c), for which reproducible and statistically solid data could be obtained. This is particularly important considering the large variability in individual patterns of kitten vocalization. Additionally, phenomena such as subharmonics and biphonation (Fig. 1g–i), occasionally observed with isolation calls (Wilden et al. 1998), may affect estimates of vocalization parameters if sample sizes are small. Although the analysis of other vocalizations might provide a more complex picture of the acoustic behavior of cats, other calls are much more difficult to evoke in a controlled manner. The present study therefore focused solely on isolation calls. Theoretically, it is possible that other vocalizations show a different pattern of dependence on age and hearing status.

The few first weeks of postnatal life are important for the kitten-–mother bond. While kittens already vocalize in the first days of life, we started with data collection from the age of 1 month to prevent the kittens undergoing a lengthy separation from their mother in the first weeks after birth.

In the present study, the investigated vocalization was referred to as the isolation call (‘meow’). This was due to the context in which the vocalization has been elicited: the animal being unrestrained but socially isolated. However, the behavioral role of this call may change throughout early development in the cat (Brown et al. 1978; Ehret 1980; Turner and Bateson 2000). The present data support this notion by the demonstration of a developmental sequence in the calls’ acoustic structure in hearing controls (Fig. 2).

The present experiments included congenitally deaf animals, whereas previous studies employed animals deafened by medical interventions. The latter have the advantage of using animals with the same social and genetic background as the hearing controls. However, surgical intervention and medical treatment may also affect other body functions, including the well-being (leading to differences in affect) and non-auditory development of the animals. The white cats, on the other hand, may have a different genetic and social background, which are potential confounding factors. To rule out these effects, we also investigated mildly hearing impaired animals of the same white-cat colony. In all grand mean comparisons (Figs. 4 and 6), the mildly hearing-impaired white cats were not different from controls. This rules out the possibility that the different social groups and the genetic background were responsible for the effects observed in deaf cats. In the detailed comparison of the developmental timeline, however, they showed some differences (Figs. 5 and 7). Hearing-impaired animals exhibited the greatest variability of F0, and this may be the underlying reason for some further differences compared with hearing cats.

The most equivocal measure in the present study is loudness. Obviously, this measure depends on the exact position and orientation of the cat’s articulatory organs. As this is impossible to precisely control in the present setting, the data may be influenced by a difference in position and have to be considered with caution. We assume, however, that these variations were similar between the groups.

Finally, the behavioral importance of vocalizations will be also dependent on the behavioral reaction of the mothers; as the mothers of the deaf cats were also deaf, some of the observations might rather be attributable to the hearing state of the mother than to the hearing of the kitten. Nonetheless, previous studies (see below) reported louder, longer and more variable vocalizations in deafened kittens (of hearing mothers: see Shipley et al. 1988, 1991), and thus it appears more plausible that the observations were due to the hearing status of the kitten.

### Discussion of results

The isolation call investigated here corresponds well to the calls described in previous studies (Brown et al. 1978; Haskins 1979; Farley et al. 1992; Romand and Ehret 1984; Shipley et al. 1988, 1991; Nicastro and Owren 2003; Scheumann et al. 2012). The fundamental frequency and the duration of these calls fall in the range of previously reported results, considering that the present data were obtained from kittens at the age of 1–3 months (Moelk 1944; Haskins 1979; Nicastro and Owren 2003).

As in previous investigations, great variability in calls was observed in the present study. However, the use of grand mean spectrograms allowed us to compare the vocalization structure at different ages. This analysis, tracing the developmental changes of acoustic structure in each individual animal, confirmed a previous suggestion (Brown et al. 1978) that the acoustic structure of the isolation call changes in the first postnatal months (Fig. 2). In all groups, irrespective of hearing status, we observed a decline in fundamental frequency with age. This effect likely reflects anatomical maturation of the vocalization apparatus. Furthermore, the present study observed a gradual age-related decrease in the juvenile initial FM component at the onset of the isolation call. These components were discernible in the first 1.0–1.5 months in hearing controls and disappeared at 2 months. The hearing-impaired animals, despite greater variability, had a mature-like isolation call at 3 months. However, in the deaf cats, the developmental sequence was decelerated, and even at 3 months, the acoustic structure still showed the juvenile FM onset component. This finding was confirmed in the statistical analysis of harmonic ratio (Fig. 7) and suggests persistent immaturity of the call structure in deaf cats.

Consistent with the trend in a previous study (Romand and Ehret 1984), the present investigation observed significantly louder vocalizations in the deaf cats. Although the interpretation of loudness has to be treated with caution, the data at least indicate that deafness increases the loudness of vocalizations in the cat. This effect has also been observed by other authors (Shipley et al. 1988) and, despite the methodological difficulties, appears as a consistent finding. Loudness of a vocalization is an important parameter that may be used for communicating affective strength in a graded communication system (Altafullah et al. 1988). Thus, an increase in energy of the isolation call in deaf animals will be of substantial communicative importance. It has been suggested that the possible increase in loudness of vocalizations in deaf animals is related to the absence of the Lombard effect (Shipley et al. 1988; Roy et al. 2011), whereas hearing kittens automatically lower the loudness of calls in a quiet environment. Deaf kittens cannot perceive the loudness of the surroundings and therefore are incapable of such downregulation. This hypothesis, however, requires testing in a more controlled way. An alternative explanation is greater distress in deaf cats when socially isolated that may both increase loudness and duration of the calls (Scheumann et al. 2012). As several other acoustic parameters were also systematically changed during development (e.g., F1/F0, see below), the finding is unlikely to be related to different levels of distress. We consequently assume that the differences in affect are not the major cause for louder calls in deaf cats; rather, the finding indicates the importance of auditory feedback for control of loudness of feline vocal production.

A decrease in F0 in deaf cats compared to hearing cats is consistent with the findings of Romand and Ehret (1984). The decrease in F0 in young kittens reported here cannot be simply explained by an increase in loudness of the calls: that would predict (as a consequence of an increased exhale pressure) an increase in F0 instead of a decrease. Similarly, as in the previous study, the decrease in F0 was statistically significant only for the youngest ages.

The present study additionally demonstrated an increase in the harmonic ratio of the vocalizations in deafness. This is important, as the effect of age on the harmonic ratio was not directly related to changes in F0. The growing vocalization apparatus would decrease F0 (due to anatomical development of the vocal tract and vocal cords) but should less affect the harmonic ratio. Indeed, no systematic effect of age on the harmonic ratio was observable in hearing cats (Fig. 7). The harmonic ratio is mainly an indicator of the active part of the vocalization, i.e., phonation, while articulation affects more the amplitude relations of the harmonics. The generation of the vocalization in the larynx, i.e., coordinated adjustments of exhale air pressure and position and tension of the vocal cords during vocalization is the likely reason for the above observations. It is true that part of the present data could again be interpreted as indicative of more distress in deaf animals in the isolation situation (Scheumann et al. 2012). However, the effects on the harmonic ratio—in combination with the developmental sequence opposite to that of hearing controls (Fig. 7b)—supports the conclusion of a change in the acoustic structure where auditory feedback is absent.

It is interesting that the deaf cats showed the largest difference in F0 compared with hearing animals in the youngest group but not at later ages when the F0 has decreased in hearing cats. One possible explanation of lower F0 in deaf cats is that it is moved into the range where somatosensory receptors can represent vibrations in a phase-locked manner and be used to control vocalizations, particularly at the youngest ages when F0 is highest. Somatosensory receptors can detect vibrations with high precision and reliable phase-locking at least up to 1 kHz (Gottschaldt and Vahle-Hinz 1981). The possible sensors for these vibrations are likely located near their source, in the vocal apparatus. Alternatively, whiskers—particularly mystacial whiskers located close to the mouth and somatosensory receptors at and around the pinna—are potential candidates. These latter can affect activity in the auditory system even in hearing animals (Shore et al. 2000; Shore and Zhou 2006). The vibration could be propagated to these locations via tissue conduction. Somatosensory inputs are reinforced by sensory deprivation (Shore et al. 2008; Meredith and Lomber 2011). Adult congenitally deaf cats indeed show moderately increased corticocortical projections from somatosensory areas into the auditory cortex (Barone et al. 2013). Such crossmodal adaptation in the control of vocalizations, however, would lead to suboptimal outcomes, as the energy required to activate the somatosensory receptors would be higher and the control would likely be less precise compared to the natural auditory feedback. Indeed, the variability of the calls was higher and the calls were louder in deaf cats. However, the absence of developmental refinement of the acoustical structure of deaf kitten vocalizations indicates either the ineffectiveness of such reorganized mechanisms of perception or another, alternative, mechanism. Further experiments are thus required to investigate the potential somatosensory control of vocalizations in deaf cats.

Shipley et al. (1988), performing experiments on two cats neonatally deafened (by mechanical destruction of the cochlea through suction) and two hearing cats, also documented an increase in loudness of the calls in deafened cats. However, the authors reported that the fundamental frequency of the vocalizations in deafened cats was higher than that in hearing cats. In the present data, compared to hearing controls, the F0 was lower and not higher in young deaf cats. As the comparisons were performed at 1 and 3 years p.n. in Shipley et al. (1988), which was much later than in the present study, it is likely that the difference in F0 is due to the difference in age at time of comparison. However, it must be borne in mind that individual vocalizations are highly variable in animals, both in duration and also in spectral structure. Shipley et al. (1988) collected 30 vocalizations at each session, compared to ∼200–700 in the present study. The previous study did not document any systematic changes in the harmonic nature of the calls but merely a change in variability in deafness. Again, the present study documents an increase in variability of the isolation calls in deaf cats, as well as changes in harmonic ratio. The combination of these findings is interesting, as the results of Shipley et al. (1988) appear more comparable to those from hearing-impaired animals in the present study that also displayed increased variability of fundamental frequency and a tendency for an increase in F0. It is very likely that, in part, the previous findings could be attributable to experiments with relatively low numbers of vocalizations and manual processing. Given the large variability in the individual vocalizations, some results could reach the level of significance by chance in such conditions (Button et al. 2013) and significant differences might be missed.

Finally, the increase in variability of the calls in deaf cats reported here and in a previous study (Shipley et al. 1988)–but not in Romand and Ehret (1984)—appears of substantial importance, additionally supporting the role of auditory feedback in maintaining and shaping the properties of vocalizations.

The present study contributes to the current discussion on the importance of auditory feedback to define species as what are known as vocal learners or non-learners (Egnor and Hauser 2004; Arriaga and Jarvis 2013). The two groups are differentiated by factors including their susceptibility to the impact of deafening on the development of vocalizations. The presented data demonstrate that congenital deafness is an important developmental factor preferentially affecting fine-tuning and reproducibility of vocalizations. However, the effect of missing auditory input was not as detrimental as in true vocal learners (e.g., songbirds, cetaceae and humans). The cats are thus neither obligate non-learners nor advanced learners but somewhere in between, although closer to the non-learners. These findings thus support the continuity hypothesis (Grimsley et al. 2011; Arriaga et al. 2012; Petkov and Jarvis 2012; Arriaga and Jarvis 2013).

However, it should be emphasized that developmental changes in vocalizations do not directly imply learning of vocalizations (Doupe and Kuhl 1999). The maturation of vocal behavior could arise from experience-independent processes of neuronal development. The complexity of the developmental changes described in the present study and their dependence on hearing status indicate at least some auditory-feedback-dependent learning process. This could be either a gradual expression of the same process as observed in vocal learners, or, alternatively, could represent a more simple sensorimotor feedback fine-tuning mechanism that is dependent on hearing and guarantees that developmental processes finally arrive at the same mature call in all individuals (which is important for communication). This latter hypothesis remains to be tested in the future.

### Comparison with humans

Language and animal communication are different phenomena. The complex symbolic nature of language, including grammar, has not been observed in animal communication systems, despite the presence of referential calls and some level of abstraction (review in Doupe and Kuhl 1999; Pepperberg 2002; Hauser et al. 2002). The present study focused on one particular feline vocalization evoked in a particular condition (in social isolation). Therefore, the conclusions cannot be simply generalized to all communication conditions and other species. However, since congenitally deaf cats have been widely used as a model of human prelingual deafness and as the kitten isolation call is similar in structure and function to the infant cry (Newman 1985, 2007), it appears important to compare the development of animal vocalizations to human vocal prelingual development. The present study supports the connection of the vocal control and auditory input (Grimsley et al. 2011). It has been argued that, for articulation in humans, an efferent copy of the motor signal is compared to the stored pattern in the auditory system (Hickok 2012). The present study indicates that a similar process may be involved even at a lower level of processing, in controlling inborn vocalizations.

There are several documented effects of deafness on vocal behavior in humans. Increase in loudness of vocal production, including speech, is one common finding (Nickerson 1975) corresponding to the present findings in cats. The vocal development of prelingually deaf children is also characterized by an increase in variance (Maskarinec et al. 1981; Seifert et al. 2002; Hocevar-Boltezar et al. 2006), similar to the present findings in deaf kittens. However, no—or only minute—changes in fundamental frequency (in the range of 2.5 %) of deaf children were observed (Campisi 2006; Campisi et al. 2006; Hocevar-Boltezar et al. 2006; cf. Scheiner et al. 2006). The absent or small effect on F0 in children (compared with larger effects in kittens) may be related to the much lower F0 in humans that is already within the range of somatosensory receptors and thus changes in F0 would not further support the control of vocal apparatus. When laughter was analyzed in deaf and hearing subjects, laughs were found to be of longer duration in the former (Makagon et al. 2008); however, laughs were less loud in deaf than in hearing subjects. Finally, one important effect of deafness on human vocal development is delayed or absent canonical babbling (Eilers and Oller 1994), corresponding to the presented findings of delayed or absent development of vocalization structure in the first 3 postnatal months. This observation in humans is of cardinal importance; however, it is more related to language development than to non-linguistic vocalizations as described here. In total, some of the present observations in cats correspond to findings regarding human vocal behavior in those with hearing loss.

## Conclusion

The present study provides automated statistical evaluation of a large set of vocalizations in hearing-impaired and deaf kittens and confirms that kittens use auditory feedback for controlling vocalizations. It shows that, despite the general preservation of isolation calls in deaf cats, the structure and the developmental pattern of the calls were affected by hearing loss. Finally, it demonstrates differential effects of mild hearing loss and complete deafness on vocalizations. Hearing-impaired animals exhibited a large variation in the acoustic structure of the calls, whereas deaf animals showed decelerated development and persistent immaturity in the acoustic structure of the calls.

As several ontogenetic changes in acoustic structure were similar for all kittens irrespective of hearing status, the results suggest that both the vocal apparatus and its neuronal motor control are subject to maturational processes, whereas the latter is additionally dependent on auditory feedback even in vocal non-learners (cats).
